# Comparative outcomes of swing segment revisions of radiocephalic arteriovenous fistula between endovascular and surgical approaches

**DOI:** 10.1371/journal.pone.0337419

**Published:** 2025-11-21

**Authors:** Suehyun Park, Sangho Lee, Hyeon Ju Kim, Hyung-Kee Kim, Seung Huh, Deokbi Hwang

**Affiliations:** 1 Division of Vascular Surgery, Department of Surgery, Kyungpook National University Hospital, Daegu, Republic of Korea; 2 Division of Vascular Surgery, Department of Surgery, Kyungpook National University Chilgok Hospital, Daegu, Republic of Korea; 3 Department of Surgery, School of Medicine, Kyungpook National University, Daegu, Republic of Korea; Ataturk University Faculty of Medicine, TÜRKIYE

## Abstract

**Objective:**

Regarding revision of vascular access (VA), endovascular methods are commonly employed owing to their procedural simplicity, yet their durability remains uncertain. This study aimed to compare clinical outcomes of swing segment (SwS) revision of radiocephalic arteriovenous fistula (RC-AVF) between endovascular and surgical approaches.

**Materials and methods:**

A retrospective cohort study comparing two groups was conducted at one tertiary hospital in South Korea. A total of 131 patients underwent endovascular or surgical revision of SwS in RC-AVF for the first time after AVF creation between 2016 and 2023. Endovascular and surgical revisions were performed in 114 and 17 patients, respectively (interposition, n = 10; patch angioplasty, n = 5; transposition, n = 1; proximalization, n = 1). Kaplan-Meier survival analysis was used to assess post-intervention primary patency (PP) and post-intervention secondary patency (SP). Multivariable Cox regression analysis was performed to adjust for potential confounders, and a subgroup analysis was conducted based on whether the SwS was in stenosis or occlusion.

**Results:**

The median minimal diameter of SwS was 1.3 mm in the endovascular group and 1.4 mm in the surgical group, and the median lesion length was 2.5 cm and 4.0 cm, respectively. Twelve-month PP was 63.5% vs 73.7% (endo vs surgical, *P* = 0.79). While PP did not differ in the stenosis subgroup, the occlusion subgroup showed significantly higher PP after surgical revision (*P* = 0.002), with surgery associated with a markedly lower risk of loss of PP events (HR 0.073).

**Conclusion:**

Surgical revision may be preferentially considered for long-segment occlusive lesions, given its superior early PP and the longer lesions typically associated with occlusions, whereas percutaneous transluminal angioplasty (PTA) remains appropriate for focal or stenotic lesions within the SwS. Consistent follow-up is essential to enable timely interventions, thereby maximizing the functionality of RC-AVF.

## Introduction

As a VA for long-term hemodialysis, AVF has been preferred due to its better durability and less complication rates than arteriovenous graft (AVG) and central venous catheter (CVC) [1,2]. Among three major types of AVF, RC-AVF used to be chosen as an initial modality, if possible [3]. Despite their better outcomes, autologous VAs including RC-AVF are not free from dysfunctions or failures. Stenosis of vein is one of the most frequent causes of AVF dysfunction [4]. The segment within 3 cm of the arteriovenous anastomosis, so-called swing segment, is known as the most common location of stenosis in RC-AVF. The stenosis of juxta-anastomotic segment, which is a part of swing segment near anastomosis, accounts for about 38 ~ 64% of all stenoses in AVF [4,5]. Considering the increasing longevity of patients and the relatively short lifespan of VAs, it is difficult to construct a new VA right away without trying to fix it when an old VA fails. Swing segment dysfunction in RC-AVF can be effectively managed through either surgical revision or endovascular intervention. The Kidney Disease Outcomes Quality Initiative (KDOQI) guideline considers it reasonable to surgically treat a failing VA after endovascular failures or when surgical outcomes are deemed markedly better [1]. However, in clinical practice, endovascular approaches such as PTA are increasingly performed at short intervals to maintain the patency of AVFs, primarily due to their procedural simplicity. However, it appears that frequent use of endovascular approaches offers a temporary rather than a fundamental solution to the problem. Thus, this study aimed to compare clinical outcomes of swing segment revision of RC-AVF between endovascular and surgical approaches.

## Materials and methods

This study was performed after obtaining approval from our Institutional Review Board (No. 2023-07-048). The research data were accessed for analysis purposes during the period from 16/08/2023–15/08/2024. Due to its retrospective design, patient informed consent was not required. Our study targeted a total of 131 patients who underwent a swing segment revision of RC-AVF for the first time after AVF creation between 2016 and 2023 in a single tertiary hospital in South Korea. While functioning RC-AVFs were included, those using the proximal radial artery (except at the wrist), non-matured fistulas, fistulas that had previously undergone balloon-assisted maturation were excluded. Balloon-assisted maturation cases were excluded because angioplasty during maturation indicates an inherently higher baseline risk of subsequent events, which could bias the comparison of post-intervention patency. Among a total of 131 fistulas created in 131 patients, 114 and 17 fistulas were repaired in an endovascular approach and a surgical approach, respectively. Data of patients’ characteristics, lesion characteristics, operation details, and the follow-up results were investigated through medical chart review. The median follow-up duration was 35.2 months (interquartile range [IQR]: 20.0–58.8 months) for all patients, 36.9 months (IQR: 24.6–76.6 months) for the surgical revision group, and 35.1 months (IQR: 18.7–56.9 months) for the endovascular revision group. Three patients were lost to follow-up despite attempts to contact them by phone. For these individuals, the last date documented in the medical records was used as the final follow-up date for analysis.

### Vascular access monitoring protocol

In the absence of observed AVF dysfunction during dialysis, a regular monitoring with physical examination was conducted at intervals of 3–6 months within one year after creation. Usually, patients were scheduled to visit the outpatient clinic two weeks and two to three months after AVF creation, three months after AVF usage, and then six months later. After that, only unstable patients in matters of hemodialysis were regularly followed up with a duplex scan. Meanwhile, if AVF could not achieve the prescribed hemodialysis smoothly, patients were referred to our clinic. Basically, the function of AVF was examined based on the arterial pressure and venous pressure during hemodialysis. When abnormal findings were detected such as weakened thrill and thrill or pulsation at abnormal sites, a duplex scan was performed for future plan.

### Indication for intervention

When the initiation or continuation of hemodialysis became difficult due to recurrent stenosis or occlusion of the swing segment, an intervention was performed. The indications for intervention included the following: (1) thrombotic occlusion, (2) difficulty in cannulation due to insufficient vein distension resulting from low flow volume, (3) vein collapse even after a successful but barely achieved cannulation, or (4) frequent alarms during hemodialysis due to high negative pressure requirements.

### Definition and outcomes of interest

**Swing segment** was defined as a segment of a vein that was mobilized for anastomosis with a radial artery. According to the literature, in RC-AVF, swing segment is located from anastomosis to the point where the curve of the cephalic vein ends [6]. In this study, we designated “swing segment” as a transposed vein segment located between the arteriovenous anastomosis and approximately 5 cm distal to it in an AVF, excluding the anastomosis itself.

**Stenosis** requiring intervention was defined as more than 70% of luminal narrowing compared to a normal post-stenotic segment of the cephalic vein, as measured by duplex scan. **Occlusion** was defined as a blockage of swing segment due to thrombosis resulting in the absence of blood flow within fistula.

**Clinical success** was determined as an effective utilization of the VA for a minimum of three dialysis sessions following a treatment. **Anatomical success** was defined as residual stenosis less than 30% on final angiogram in the endovascular revision group [7,8].

**Post-intervention patency rates** were assessed according to the definition of reporting standards by Sidawy et al [8]. **Post-intervention primary patency (PP)** was defined as the interval from the first intervention, regardless of surgical or endovascular revision, to the next intervention or the time of measurement of patency. **Post-intervention assisted primary patency (APP)** was defined as the interval from the first intervention to access thrombosis or the time of measurement of patency, including all intervening similar manipulations designed to maintain the functionality of the access. **Post-intervention secondary patency (SP)** was defined as the interval from the first intervention to access abandonment or the time of measurement of patency, including similar intervening manipulation designed to reestablish functionality in thrombosed access.

### Operative details

In cases of stenosis, the method of repair between surgical and endovascular approach was determined at a surgeon’s discretion, considering the patient’s surgical history, duplex scan findings, and overall surgical tolerance. Meanwhile, with respect to thrombotic occlusion, vessel dissection and thrombectomy with Fogarty catheter after cutdown almost always came before revision of swing segment. Therefore, routinely preceded thrombectomy was not considered one of the types of surgical revisions. Both endovascular and surgical revisions were conducted under local anesthesia.

#### 1) Endovascular revision.

For balloon angioplasty, a retrograde approach was basically preferred by inserting the sheath on the proximal forearm cephalic vein. After acquiring the angiographic image, either a hydrophilic 0.035” guidewire or a 0.014” guidewire was used to pass the stenotic lesion based on the extent of stenosis. A guidewire was usually advanced into the distal radial artery through the anastomosis. However, when retrograde passage was difficult with any guidewires, an antegrade approach was very rarely attempted by puncturing a radial artery distal to anastomosis. Balloon diameter was decided according to the diameter of adjacent normal vein with 1 mm oversizing. Initially a semi-compliant balloon was selected to dilate the stenotic lesion. However, if the balloon was not fully expanded after repeated inflation, a non-compliant balloon was used. Regardless of the balloon type, the balloon was inflated up to the nominal or rated burst pressure in which the balloon was fully expanded and maintained for approximately 120–180 seconds. After undergoing balloon angioplasty at least twice, final angiography was acquired to ensure dilation of the stenosed swing segment.

#### 2) Surgical revision.

**Fig 1:**
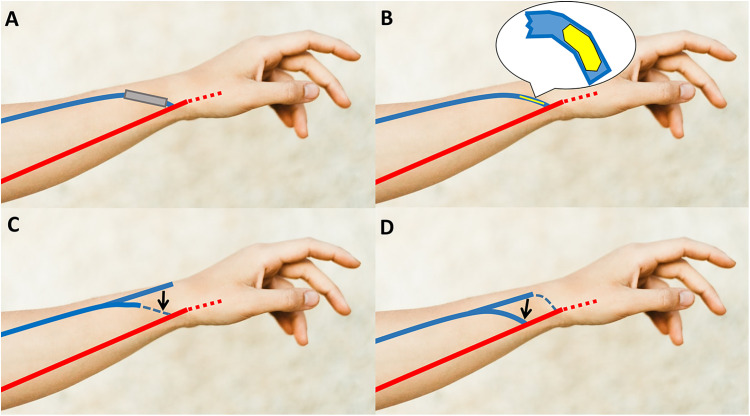
Illustration of surgical revisions for swing segment steno-occlusion in radiocephalic arteriovenous fistula: (A) Interposition, (B) Patch angioplasty, (C) Transposition, (D) Proximalization (Blue: Cephalic vein, Dashed blue: Stenotic vein segment, Red: Radial artery, Dashed red: Omitted distal artery, Yellow: Patch, Gray: Interposition graft).

One of the following four methods were used to repair the swing segment steno-occlusion (Fig 1). Surgical revision was considered, especially if the stenosis of at least longer than 2 cm was combined with diffuse thickening of vein wall. When both transposition and proximalization were inappropriate, interposition or patch angioplasty was planned based on the extent of stenosis.

2-1) Interposition

The overlying skin was usually incised along the stenosed vein and the vein was dissected up to the normally dilated segment for clamping. To replace the stenosed segment, an expanded polytetrafluoroethylene (ePTFE) graft was anastomosed with the dilated vein or radial artery at anastomosis using prolene 6−0 in an end-to-end or a side-to-end fashion (Fig 1A). Considering the anticipated vein expansion and improved flow following revision, a 6- or 7-mm interposition graft was used after spatulation of the vein and the graft to manage the size mismatch and prevent potential anastomotic stenosis.

2-2) Patch angioplasty

The incision was conducted in the same way as in the case of interposition. When the extent of stenosis and the wall thickening were not very severe, an artificial graft designed in an elliptical shape was connected with the vein after longitudinal venotomy along the swing segment. Bovine pericardial patch or ePTFE graft was usually used (Fig 1B).

2-3) Transposition

After dissection of another well-dilated forearm cephalic vein usually located lateral to the main cephalic vein and tunneling the dissected vein toward the existing anastomosis, an end-to-end anastomosis or a side-to-end anastomosis between a not-stenosed main proximal cephalic vein stump or a radial artery and the transposed cephalic vein was made, respectively. This method was possible when there was a well-dilated proper vein among several branches of the forearm cephalic vein at the wrist (Fig 1C).

2-4) Proximalization

When the cephalic vein was long enough to be utilized as ports for needling after resecting the stenotic segment, the well-dilated main cephalic vein was moved medially and newly anastomosed to the more proximal radial artery than the previous anastomosis. Previous anastomosis was ligated before a new anastomosis was made (Fig 1D).

### Statistical analysis

For continuous variable, Student’s t-test or Mann-Whitney U-test was performed after a normality test. The chi-square test or Fisher exact test was performed for categorical variables. Kaplan-Meier survival analysis was used to assess PP, APP and SP. The log-rank test was used to assess the statistically significant difference in patency between the two groups. Cox proportional hazards regression analyses were performed to identify factors associated with loss of PP in the entire cohort and in the subgroup of patients who underwent intervention for stenosis or occlusion. Hazard ratios (HRs) with 95% confidence intervals (CIs) were calculated. Variables with statistical significance in univariable analyses, as well as clinically relevant factors such as age, lesion length, and fistula age, were included in the multivariable models to adjust for potential confounding. Poisson regression within a generalized linear model was used to evaluate the relationship between reoperation rates and revision types. All statistical analyses were performed using IBM SPSS statistics (v.25.0; IBM Corp., Armonk, NY, USA) and statistical significance was considered at *P* < 0.05.

## Results

A total of 131 patients underwent swing segment revision in RC-AVF during the study period. Their mean age was 66.0 years old. Male patients accounted for 66.4%. An endovascular revision was performed for 114 patients and a surgical revision was performed for 17 patients, comprising 10 cases of interposition, 5 cases of patch angioplasty, one case of transposition, and one case of proximalization. There were no significant differences in patient characteristics between surgical and endovascular revision groups, although the endovascular group showed a trend toward older age (*P* = 0.058). Regarding etiology, diabetes mellitus was the most common cause of renal failure, followed by hypertension and chronic glomerulonephritis (Table 1).

**Table 1:** Characteristic of patients who underwent swing segment revision of radiocephalic arteriovenous fistula (RC-AVF).

Variable	Total (n = 131)	Endo (n = 114)	Surgery (n = 17)	*P-*value
**Age – years**	66.0 (53.0-73.0)	67.0 (53.0-74.0)	56.0 (42.0-71.5)	0.058
**Male**	87 (66.4)	73 (64.0)	14 (82.4)	0.14
**Comorbidities**				
**Hypertension**	126 (96.2)	110 (96.5)	16 (94.1)	0.51
**Diabetes mellitus**	82 (62.6)	71 (62.3)	11 (64.7)	0.85
**Coronary artery disease**	20 (15.3)	18 (15.8)	2 (11.8)	> 0.99
**Congestive heart failure**	17 (13.0)	15 (13.2)	2 (11.8)	> 0.99
**Arrhythmia**	4 (3.1)	4 (3.5)	0 (0.0)	> 0.99
**Cerebrovascular disease**	12 (9.2)	10 (8.8)	2 (11.8)	0.66
**CCVD**	30 (22.9)	26 (22.8)	4 (23.5)	> 0.99
**Dyslipidemia**	70 (53.4)	59 (51.8)	11 (64.7)	0.32
**Etiology of ESRD**				
**Hypertension**	23 (17.6)	19 (16.7)	4 (23.5)	0.50
**Diabetes mellitus**	72 (55.0)	64 (56.1)	8 (47.1)	0.48
**CGN**	26 (19.8)	23 (20.2)	3 (17.6)	> 0.99
**PCKD**	4 (3.1)	4 (3.5)	0 (0.0)	> 0.99
**Others**	5 (3.8)	3 (2.6)	2 (11.8)	0.13
**Unknown**	1 (0.8)	1 (0.9)	0 (0.0)	> 0.99

Note: Data are presented as n (%), median (Interquartile range).

ESRD, end-stage renal disease; CGN, chronic glomerular nephritis; PCKD, polycystic kidney disease; CCVD, cardiocerebrovascular disease (Coronary artery disease + Cerebrovascular disease).

Lesion characteristics are described in Table 2. The median length of stenotic lesion was 2.5 cm (IQR: 1.5–4.0 cm) in the endovascular revision group and 4.0 cm (IQR: 2.5–5.0 cm) in the surgical revision group (*P* = 0.008). The median diameter of swing segment was 1.4 mm (IQR: 0.9–1.7 mm) in the surgical revision group and 1.3 mm (IQR: 1.0–1.6 mm) in the endovascular revision group (*P* = 0.58). While the average age of RC-AVF at initial intervention was 44.4 months in the surgical revision group, it was 8.8 months in the endovascular revision group (*P* = 0.001). While 93.0% of endovascular revisions were conducted because of stenosis, 64.7% of surgical revisions were made because of occlusion (*P* = 0.001). Diameters of cephalic vein and radial artery before AVF creation showed no significant difference between surgical and endovascular revision groups. Minimal diameter of cephalic vein was 2.6 ± 0.4 mm in the surgical revision group and 2.5 ± 0.5 mm in the endovascular revision group (*P* = 0.56). Radial artery diameter was 2.4 ± 0.5 mm in the surgical revision group and 2.4 ± 0.4 mm in the endovascular revision group (*P* = 0.90).

**Table 2:** Lesion characteristics of swing segment.

Variable	Total (n = 131)	Endo (n = 114)	Surgery (n = 17)	*P-*value
**When revision**				
**Lesion length (cm)**	3.0 (2.0–4.0)	2.5 (1.5–4.0)	4.0 (2.5–5.0)	**0.008**
**Minimal diameter (mm)**	1.3 (1.0–1.6)	1.3 (1.0–1.6)	1.4 (0.9–1.7)	0.58
**Fistula age (month)**	11.6 (4.9-22.6)	8.8 (4.7-15.0)	44.4(17.7-98.0)	**0.001**
**When AVF formation (mm)**				
**Minimal diameter, cephalic vein**	2.5 (±0.5)	2.5 (±0.5)	2.6 (±0.4)	0.56
**Maximal diameter, cephalic vein**	3.4 (±0.7)	3.5 (±0.7)	3.3 (±0.4)	0.31
**Radial artery diameter**	2.4 (±0.4)	2.4 (±0.4)	2.4 (±0.5)	0.90
**Stenosis vs. Occlusion**				**0.001**
**Stenosis**	112 (85.5)	106 (93.0)	6 (35.3)	
**Occlusion**	19 (14.5)	8 (7.0)	11 (64.7)	
**Lesion length of subgroup (cm)**				
**Stenosis**	2.5 (1.6–4.0)	2.5 (1.5–4.0)	2.5 (2.4–4.3)	0.52
**Occlusion**	4.0 (3.0–5.0)	3.0 (2.0–5.0)	4.0 (3.0–5.0)	0.27

Note: Data are presented as n (%), median (Interquartile range), or mean ± standard deviation. P-values <0.05 are considered statistically significant and bolded.

All lesion lengths and diameters of the swing segment, cephalic vein, and radial artery were measured using ultrasonography.

While there was no complication in the surgical revision group, 7 cases had complications in the endovascular revision group. Out of these 7 cases, vein rupture occurred in 5 cases during balloon angioplasty. After immediate balloon tamponade, no additional extravasation was observed on final angiogram. In two cases with thrombosis occurring during prolonged ballooning, thrombectomy and additional balloon angioplasty were performed. Clinical success rate was 100% in the surgical revision group and 95.6% (109/114) in the endovascular revision group. Anatomical success rate was 100% in the surgical revision group and 98.2% (112/114) in the endovascular revision group. Despite residual stenosis, endovascular procedure was finished because thrill was improved. In the two cases with an anatomical failure (defined as residual stenosis more than 30%), VAs functioned effectively for hemodialysis.

**Fig 2:**
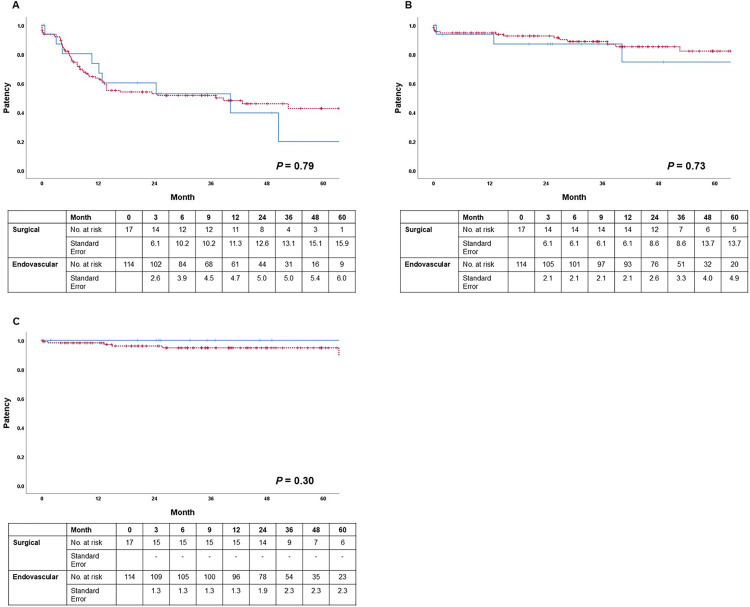
Comparison of patency rates among the entire cohort of patients across swing segment revision groups: endovascular revision vs surgical revision. **(A)** Primary patency, **(B)** Assisted primary patency, and **(C)** Secondary patency are depicted. The endovascular revision group is shown as a *dashed line* and the surgical revision group as a *solid line*. Crosses (+) indicate censored observations, meaning that the patient’s patency status was not observed beyond that time point.

In Kaplan-Meier survival analysis, there was no significant difference in PP, APP, or SP between the two groups (PP, *P* = 0.79; APP, *P* = 0.73; SP, *P* = 0.30) (Fig 2). PP rates at 3, 6, 12 months were 93.8%, 80.4%, 73.7% in the surgical revision group and 92.0%, 79.2%, 63.5% in the endovascular revision group, respectively. APP rates at 3, 6, 12 months were all 93.8% in the surgical revision group and were all 94.7% in the endovascular revision group, respectively. In the surgical revision group, no VA was abandoned during the study period. SP rates at 1, 2, 3 years were 98.2%, 96.1%, 94.8% in the endovascular revision group, respectively.

Multivariable analysis including age, sex, comorbidities, lesion characteristics (minimum diameter and lesion length), fistula age, and treatment modality (endovascular vs. surgical) showed that only age and lesion length were independently associated with loss of PP (Table 3). Older age was associated with a decreased risk of PP loss (HR 0.977, 95% CI: 0.959–0.995, *P* = 0.011), whereas longer lesion length was associated with an increased risk (HR 1.249, 95% CI: 1.030–1.515, *P* = 0.024). After adjustment, fistula age and treatment modality, which were significantly different between groups in Table 2, were no longer associated with outcome in the overall cohort.

**Table 3:** Factors associated with loss of primary patency in whole cohort.

Variable	Univariable analysis		Multivariable analysis	
	HR (95% CI)	*P*-value	aHR (95% CI)	*P*-value
**Age**	0.980 (0.963–0.997)	**0.023**	0.977 (0.959–0.995)	**0.011**
**Sex**	1.068 (0.640–1.780)	0.802		
**HTN**	0.652 (0.236–1.796)	0.408		
**DM**	0.921 (0.559–1.517)	0.747		
**CGN**	0.821 (0.446–1.511)	0.527		
**CAD**	0.558 (0.224–1.392)	0.211		
**CHF**	0.799 (0.345–1.853)	0.601		
**Arrhythmia**	2.149 (0.668–6.907)	0.199		
**CVD**	1.136 (0.455–2.838)	0.785		
**CCVD**	0.759 (0.386–1.492)	0.424		
**Dyslipidemia**	1.137 (0.695–1.859)	0.609		
**Lesion length**	1.221 (1.019–1.463)	**0.031**	1.249 (1.030–1.515)	**0.024**
**Minimal diameter**	0.841 (0.490–1.444)	0.530		
**Fistula age**	0.999 (0.992–1.006)	0.764	0.996 (0.986–1.005)	0.387
**Approach**				
**Endo**	Ref(1)			
**Surgery**	1.225 (0.624–2.408)	0.556	1.118 (0.486–2.570)	0.793

CGN, chronic glomerulonephritis; CAD, coronary artery disease; CCVD, cerebro-cardiovascular disease (CAD + CVD); CHF, congestive heart failure; CI, confidence interval; CVD, cerebrovascular disease; DM, diabetes mellitus; HTN, hypertension; aHR, adjusted hazard ratio.

**Fig 3:**
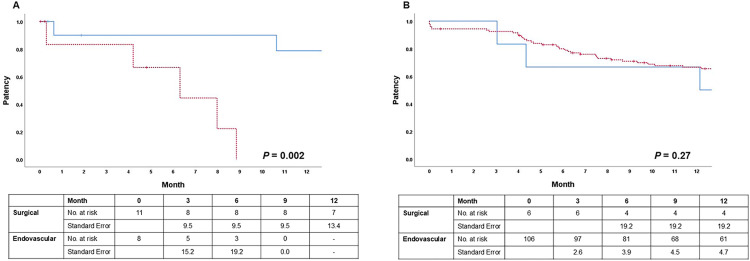
Comparison of primary patency rates within the (A) occlusion arm and (B) stenosis arm between the two swing segment revision groups: endovascular revision group vs surgical revision group. The endovascular revision group is shown as a *dashed line* and the surgical revision group as a *solid line*. Crosses (+) indicate censored observations, meaning that the patient’s patency status was not observed beyond that time point.

To assess whether the effect of treatment modality on PP differed between stenosis and occlusion, an interaction test was performed after adjustment for age, lesion length, and fistula age. This analysis demonstrated a significant interaction (*P* = 0.016). Accordingly, subgroup analyses were performed according to whether the swing segment was in stenosis or occlusion (Table 4). In these models, fistula age was not significantly associated with PP in either subgroup. In the stenosis group, treatment modality was not associated with outcome, whereas in the occlusion group it had a significant impact, with surgical revision being associated with a markedly lower risk of loss of PP events (HR 0.073). In contrast to the occlusion group, age and lesion length were significant predictors in the stenosis group; longer lesion length was associated with poorer outcomes, whereas older age had a protective effect. In Kaplan-Meier survival analysis, surgical revision was associated with better PP than endovascular revision in the occlusion group (*P* = 0.002, Fig 3A). In contrast, in the stenosis group, PP appeared similar between the two groups for up to 12 months (*P* = 0.27, Fig 3B). Reoperation rates after initial revision were 0.22 and 0.24 events/AVF-year in surgical and endovascular revision groups, respectively (*P* = 0.71). This lack of difference was also evident in the occlusion (*P* = 0.45) and stenosis (*P* = 0.72) subgroups.

**Table 4:** Factors associated with loss of primary patency within subgroup of stenosis or occlusion.

Variable	Stenosis		Occlusion	
	aHR (95% CI)	*P*-value	aHR (95% CI)	*P*-value
**Age**	0.975 (0.955–0.994)	**0.012**	0.956 (0.905–1.010)	0.107
**Lesion length**	1.271 (1.027–1.573)	**0.027**	1.172 (0.679–2.022)	0.568
**Fistula age**	0.986 (0.969–1.003)	0.103	1.001 (0.988–1.014)	0.918
**Approach (Surgery)**	2.512 (0.750–8.413)	0.135	0.073 (0.008–0.643)	**0.018**

aHR, adjusted Hazard ratio.

Multivariable Cox regression models were adjusted for age, lesion length, fistula age, and treatment modality (surgery vs. endovascular).

## Discussion

With rising aging population and growing prevalence of diabetes mellitus and cardiovascular disease, end-stage renal disease population requiring hemodialysis will continue to increase unless the supply of kidneys for transplantation catches up with the demand. Besides quality of life and cost benefits, AVFs reportedly take precedence over AVGs or CVCs because of their lower rates of morbidity and mortality [9–12]. Considering increased longevity of human and finite life of VA, it appears that fistula first strategy will still work and RC-AVF at wrist will be considered the first choice. However, swing segment stenosis of RC-AVF is a hardship to overcome for its prolonged use. Previous studies have compared outcomes between surgical and endovascular approaches in juxta-anastomotic stenosis in RC-AVF [13–17]. Long et al. have found better PP in surgical revision compared to endovascular revision but no significant difference in PP or APP between the two groups when the stenosis is confined to the vein without involving anastomosis [14]. Contrary to their study, our study dealt with RC-AVFs presenting venous stenosis primarily, not a significant anastomotic or arterial stenosis. Our study also showed similar results (overall PP, APP, SP) in surgical and endovascular revision groups. However, our results were probably due to different lesion characteristics between the two groups, unlike the study of Long et al., in which the two treatment groups had similar length of stenosis. In contrast, our study revealed that in the entire cohort of patients, the lesion length was significantly shorter in the endovascular revision group, likely attributed to a longer stenosis in the surgical group within the occlusion arm. These results, which would have been clearly superior in the surgical revision group if the lesion length had been similar, might have been offset by more severe lesion characteristics in the surgical revision group, resulting in similar outcomes. In fact, within the occlusion arm, the surgical group showed better PP than the endovascular revision group and the lesion length was similar between the two groups within the stenosis arm. This finding is concordant with previous studies [18,19]. In the same context, unlike the surgical group, where issues arose from sites other than the swing segment, all five clinical failures in the endovascular treatment group were attributed to restenosis in the swing segment, initially presenting with a 3–4 cm stenosis. Notably, these cases were successfully resolved through surgical intervention. However, contrary to the findings of Tessitore et al., who reported higher restenosis rates in the endovascular treatment group than in the surgical revision group [17], our study showed no significant difference in reoperation rates between surgical and endovascular revisions or between stenosis and occlusion subgroups. This may be partly explained by the fact that patients did not consistently adhere to a single treatment modality; subsequent reinterventions were performed using either approach as clinically indicated.

A significant limitation of this study is the lack of well-matched characteristics between the two groups, particularly regarding the total number of patients, age, fistula duration, and lesion characteristics such as lesion length. This discrepancy raises concerns about potential selection bias. Although fistula duration differed between the two groups, the majority of cases in both groups had been in place for over a year, indicating that the fistulas were already in a sufficiently matured state. Thus, emphasizing fistula duration in terms of maturation may not carry additional significance. Notably, although fistula age differed between the two groups, it was not associated with outcome in the multivariable analysis after adjustment for age, lesion length, and treatment modality. On one hand, considering advanced fistula aging and higher lesion severity of RC-AVF, patients in the surgical revision group might not have followed the program as strictly as those in the endovascular revision group. On the other hand, it is possible that they did not visit the clinic because there were no problems related to hemodialysis, indicating excellent durability of surgical revision.

As mentioned in the results, there was a significant difference in lesion characteristics, particularly lesion length, between the two groups, which influenced the choice of surgical method. However, lesion length is a critical factor, with existing evidence suggesting, or at least raising controversy, that surgical revision may achieve better outcomes for longer lesions. Assigning even longer lesions to endovascular revision purely for the sake of randomization or balanced distribution would be ethically questionable. Therefore, treatment allocation was determined at the operator’s discretion, based on experience and established knowledge from prior literature. This highlights why a retrospective design was unavoidable. As with any retrospective study, the influence of selection bias cannot be entirely ruled out. The multivariable analysis indicated that the effect of age and lesion length on PP in the overall cohort (Table 3) was primarily attributable to the stenosis subgroup (Table 4), which exhibited a similar trend. The finding that older age was associated with a lower risk of adverse outcomes may be explained by treatment selection bias. In elderly patients, whose general condition is often poorer than that of younger individuals, conservative management rather than intervention was likely preferred even for similar lesions. In addition, their shorter life expectancy may have resulted in a higher likelihood of death during follow-up, leading to a shorter observation period and fewer recorded events. Lower physical activity and reduced arm use of the access arm in older individuals may also contribute, although this remains speculative and requires further investigation.

Nevertheless, the findings of this study provide useful insights into clinical decision-making, while acknowledging the inherent limitations of its design. Despite the small sample size, our study reflects real-world practice by incorporating various revision techniques, including transposition, interposition, and patch angioplasty, as well as proximalization. In most cases (15/17), interposition or patch angioplasty was performed instead of proximal relocation of the arteriovenous anastomosis. This decision was primarily due to concerns about maintaining adequate cannulation length after the revision of a considerably long lesion. Although the ESVS guidelines recommend proximal relocation of the anastomosis for juxta-anastomotic stenosis, these anatomical challenges influenced the choice of technique [20]. Nevertheless, the relatively small number of surgical patients limited subgroup analyses and residual unmeasured confounding cannot be excluded. In addition, the difference in follow-up duration between the groups may have influenced the interpretation of long-term outcomes and should be acknowledged as a limitation of this study. Future studies with larger cohorts and prospective or randomized controlled trials are warranted to validate our findings.

Meanwhile, the impact of the timing of stenosis recurrence on the choice of treatment modality should also be considered. In cases where stenosis recurs shortly after PTA, surgical revision may eventually be required, even if the lesion is not particularly long. Such cases often involve a history of multiple PTA procedures and are accompanied by significant wall thickening. However, this study focused on outcomes after the first single revision—whether endovascular or surgical—following AVF creation, thereby minimizing potential bias to some extent.

Endovascular treatment has been more commonly performed than surgical revision, which is a phenomenon not unique to our hospital. This trend can be attributed to procedural convenience and a relatively high cost associated with PTA, particularly within the healthcare system in South Korea. While population-based reimbursement models are increasingly explored worldwide, Korea’s healthcare reimbursement system primarily follows a Fee-for-Service structure. In Korea, the procedure fee for endovascular intervention is approximately 1.7–3.5 times higher than that for surgical revision, indicating a substantially higher initial cost (S1 Table). As a result, these procedures are predominantly performed by non-surgical specialists, and it is common for PTA to be used until no endovascular alternatives remain. At this point, the opportunity for a simpler surgical solution might have already been missed. From both the cost-effectiveness and the patient’s quality of life perspectives, this delayed transition or wrong choice of revision methods is indeed disadvantageous. Thus, in the context of Fee-for-Service structure like Korea, the endovascular group appears to impose greater psychosocial and economic burdens from the patient’s perspective. Although a formal cost-effectiveness analysis could not be performed because of the retrospective design and the heterogeneous patterns of subsequent reinterventions, a prospective study with a standardized protocol will be necessary to clarify the true cost-effectiveness of surgical versus endovascular approaches. However, because procedure reimbursements in Korea are substantially lower than those in Western countries, the absolute cost values presented in this study may not be directly comparable to those reported from other healthcare systems.

However, endovascular revision should not be solely seen as negative. Despite its lower long-term patency rates, it offers a less invasive option that is beneficial for patients in poor condition. Furthermore, recent introduction of drug-coated balloons (DCBs) in VA procedures raises the possibility of improved outcomes. However, most available evidence has focused on venous outflow lesions, including the cephalic arch, and substantial heterogeneity among studies persists [21–23]. Unfortunately, DCBs were not yet approved for clinical use in our country during the study period. Future investigations specifically targeting the swing segment of autologous VAs are warranted to elucidate whether DCBs can achieve superior or at least comparable early outcomes compared with surgical revision for swing segment steno-occlusion. Such studies may herald a new phase in the management of VA swing segments.

## Conclusions

In conclusion, surgical revision may be preferentially considered for long-segment occlusive lesions, given its superior early PP and the longer lesions typically associated with occlusions, whereas PTA remains appropriate for focal or stenotic lesions within the swing segment, where the surgical benefit is limited. Consistent follow-up is also crucial to prevent rapid patency decline and enable timely repairs during stenotic states, thus prolonging the functionality of RC-AVF. Future studies are warranted to refine treatment algorithms and optimize patient outcomes.

## Supporting information

S1 Table Summary of procedure-related costs and reoperation rates. KRW: Korean Won. Surgical costs varied depending on the use of patch or graft materials. Costs represent approximate institutional billing data. *For reference, 1 USD ≈ 1,350 KRW (as of mid-2025). Procedure reimbursements under the Korean national insurance system are generally lower than those in Western countries.*
(PDF)

## Abbreviations

VA: Vascular Access
AVF: Arteriovenous fistula
RC-AVF: Radiocephalic Arteriovenous fistula
PP: Post-intervention primary patency
APP: Post-intervention assisted primary patency
SP: Post-intervention secondary patency
PTA: Percutaneous transluminal angioplasty
DCBs: Drug-coated balloons
