# Supplementary material for: Comparative outcomes of swing segment revisions of radiocephalic arteriovenous fistula between endovascular and surgical approaches
Source: PLoS One. 2025 Nov 21;20(11):e0337419. doi: 10.1371/journal.pone.0337419 (PMC12637910; doi:10.1371/journal.pone.0337419)
Supplement: S1 Table — KRW: Korean Won. Surgical costs varied depending on the use of patch or graft materials. Costs represent approximate institutional billing data. For reference, 1 USD ≈ 1,350 KRW (as of mid-2025). Procedure reimbursements under the Korean national insurance system are generally lower than those in Western countries. (PDF) [file pone.0337419.s001.pdf]

S1 Table. Summary of procedure-related costs and reoperation rates.

Category	Surgical revision	Endovascular revision
**Approximate procedure cost (KRW)**	677,780 (no patch/graft)<br>807,322 (patch angioplasty)<br>1,394,515 (interposition graft)	2,409,715
**Reoperation rate**		
**Overall**	0.22	0.24
**Occlusion subgroup**	0.19	0.28
**Stenosis subgroup**	0.28	0.24

KRW: Korean Won. Surgical costs varied depending on the use of patch or graft materials. Costs represent approximate institutional billing data.

For reference, 1 USD ≈ 1,350 KRW (as of mid-2025). Procedure reimbursements under the Korean national insurance system are generally lower than those in Western countries.
